# Diagnostic accuracy of doctors at the emergency department and radiologists in differentiating between complicated and uncomplicated acute appendicitis

**DOI:** 10.1007/s00068-023-02442-2

**Published:** 2024-01-17

**Authors:** Jochem C G Scheijmans, Wouter J Bom, Rabia S Deniz, Anna A W van Geloven, Marja A Boermeester, The SAS Collaborative, F Alberts, S MA Bachiriden Bakker, B Bisschops, E Boersma, M D M Bolmers, W M Bosman, H Bril, C Buurman, E F W Courrech Staal, P Davids, R Detering, M G W Dijkgraaf, B L Dijkstra, P Drillenburg, A Dinaux, P van Duijvendijk, W J van Eden, R Franken, S Gans, M Gaspersz, A M van Geel, M F Gerhards, H Ghori, J W C Gratama, I Groenendijk, P Hellebrekers, A van Hemert, M Henebiens, H Heydari, K in ’t Hof, T J Hoogteijling, P M Huisman, G van Ingen, S Jensch, A M Jonker, F Joosten, L Koens, N Kraaijvanger, K C Kuijpers, T Y S Le Large, D Linzel, M H J Loos, A M F Lopes Cardozo, L B Meijer-Jorna, M Mulder, N Mullaart, S J Oosterling, J Oudejans, T Pappot, S Peeters, C Pleiter, MA de Roos, C Rosman, C C van Rossem, M M Scheurkogel, L Scholten, T Schut, J Stoker, O W T Tiddens, S Ubels, F E E de Vries, L F J Walraven, E M A Wiegerinck, J K Wiggers, M Witt, N Wolfhagen, L van de Wouw

**Affiliations:** 1 Department of Surgery, Amsterdam University Medical Center, University of Amsterdam, Amsterdam, The Netherlands; 2 Department of Surgery, Tergooi Medical Center, Hilversum, The Netherlands; 3 Amsterdam Gastroenterology Endocrinology and Metabolism, Amsterdam, The Netherlands

**Keywords:** Acute appendicitis, Uncomplicated acute appendicitis, Complicated acute appendicitis, Diagnostic accuracy, Doctor’s judgement

## Abstract

**Purpose:**

To determine the accuracy of final judgements of doctors at the emergency department (ED) and radiologists to differentiate between complicated and uncomplicated acute appendicitis, because these have different treatment options.

**Methods:**

This prospective, multicenter study included adult patients with imaging-confirmed acute appendicitis, operated with intention to appendectomy. Both doctors at ED and radiologists assessed appendicitis severity as a final judgement of “uncomplicated” or “complicated” appendicitis. Doctors at ED integrated clinical, laboratory, and imaging findings. Radiologists relied solely on imaging findings. Outcomes were accuracy of these judgements for diagnosis of complicated appendicitis compared to the reference standard by an adjudication committee.

**Results:**

After imaging, 1070 patients with confirmed acute appendicitis were included. Doctors at ED accurately labelled 656 of 701 (93.6%) patients with true uncomplicated appendicitis as uncomplicated, and 163 of 369 (44.2%) patients with true complicated appendicitis were labelled as complicated. Sensitivity, specificity, and positive and negative predictive values (PPV and NPV) for complicated appendicitis were 44.2%, 93.6%, and 78.4% and 76.1%, respectively. Comparable accuracy was found for the radiologist’s assessment in 941 patients, with true positive rates of 92.2% (581 of 630 patients) for uncomplicated appendicitis and 46.6% (145 of 311 patients) for complicated appendicitis.

**Conclusion:**

More than half of all patients with true complicated appendicitis is incorrectly classified as uncomplicated appendicitis according to the judgements of doctors at ED, integrating clinical, laboratory, and imaging results, and of radiologists assessing diagnostic imaging. These judgements are thereby not sufficiently reliable in ruling out complicated appendicitis.

**Supplementary Information:**

The online version contains supplementary material available at 10.1007/s00068-023-02442-2.

## Introduction

Worldwide, acute appendicitis is one of the most common surgical emergencies [1]. The diagnostic goal is shifting from correct identification of patients with acute appendicitis to differentiation between complicated and uncomplicated appendicitis. In contrast to complicated appendicitis, uncomplicated appendicitis does not require emergency surgery, since this form of acute appendicitis can be treated by antibiotics [2–5] or semi-emergent appendectomy, or may even resolve spontaneously [6]. Therefore, reliable exclusion of complicated appendicitis is of paramount importance in the assessment of the appendicitis severity [7].

Several methods have been described to distinguish between complicated and uncomplicated appendicitis [7]. While imaging modalities have demonstrated efficacy in diagnosis of acute appendicitis, imaging alone falls short in ruling out complicated appendicitis [7, 8]. Models that combine imaging findings with clinical variables seem more promising [7, 9, 10]. However, these models do not take into account any subjective judgement of the doctor’s interpretation of laboratory and imaging findings. This doctor’s judgement, based on experience, clinical perception, and intuition, has shown high negative predictive values (NPVs, 96–99%) for the diagnosis of acute appendicitis in children or adults before imaging [11–13]. Nevertheless, none of these studies has investigated this judgement in the context of distinguishing between complicated and uncomplicated appendicitis, let alone this doctor’s final judgement when integrating their interpretation of laboratory and imaging findings.

Kim et al. explored the accuracy of radiologists’ judgement for the differentiation between complicated and uncomplicated appendicitis. They reported a pooled sensitivity and specificity for complicated appendicitis of 64% and 76%, respectively [14]. However, radiologists typically do not evaluate the patient directly. It could be argued that the judgement of the attending doctor at the emergency department (ED), who has extensively examined the patient, may be more accurate with respect to the differentiation between complicated and uncomplicated appendicitis, but literature on this subject is currently lacking.

The aim of the present study was to determine the accuracy of the doctor’s judgement integrating all available information including clinical, laboratory, and imaging results for diagnostic differentiation between complicated and uncomplicated appendicitis in the ED. The second objective was to determine the accuracy of this differentiation for the radiologist when specifically asked to indicate appendicitis severity — complicated or uncomplicated — based on imaging findings.

## Methods

A prospective, observational study, termed Scoring system of Appendicitis Severity (SAS) study, was conducted between January 2020 and August 2021 in 11 Dutch hospitals. The primary aim of the main study was to externally validate an objective scoring model designed for differentiation between complicated and uncomplicated appendicitis [9], and to develop the SAS [15]. The present study was a predefined sub-analysis of this SAS study [15]. Informed consent was obtained from all participating patients. Diagnostics and treatment were performed according to national or local guidelines. The updated list of the STARD 2015 guidelines was used in the design and implementation of the study [16].

### Study population

Consecutive adult patients (≥ 18 years) with an imaging-confirmed diagnosis of acute appendicitis and operated with intention to appendectomy were included in the SAS study cohort. Conservatively treated patients were excluded. Clinical practice dictated the type of imaging. For all patients of the SAS study cohort, the first attending doctor at the ED of each case was asked to provide an independent assessment of the appendicitis severity, in terms of a subjective final judgement of “uncomplicated” or “complicated” appendicitis. These doctors were consultant surgeons, surgical trainees, consultant emergency doctors, or emergency trainees. They had access to clinical, laboratory, and imaging results, and the assessment was completed prior to any surgery. Similarly, the radiologist of each case was asked to evaluate the severity of appendicitis as “uncomplicated” or “complicated,” but they relied solely on imaging findings. Current sub-analysis of the SAS study excluded participants from the original cohort who did not have any doctor’s and/or radiologist’s judgements of appendicitis severity.

### Data collection

Data regarding clinical, laboratory, and imaging findings were prospectively gathered through standard reports in the electronic health record by the attending doctor at the ED, the radiologist, the operating surgeon, and the pathologist. In addition to the assessment of appendicitis severity, doctors at the ED as well as radiologists were asked about their years of experience and the level of confidence in their appendicitis severity judgement. This confidence level was evaluated according to an 11-point Likert scale (score 0–10) and categorized as follows: a score of 7 or higher was defined as “certain” and a score of 6 or lower was interpreted as “uncertain.” All data were prospectively collected into the data collection program CASTOR EDC [17].

### Test definitions

The index tests were appendicitis severity according to the final judgement of the attending doctor at the ED and the assessment of the radiology imaging reader, collected as described above. The reference standard was the final diagnosis, classified as uncomplicated or complicated appendicitis, assigned by an adjudication committee based on all available data, including intraoperative and histopathological findings. The committee consisted of two surgeons, two radiologists, one pathologist, one surgeon-in-training, and one research fellow. Uncomplicated appendicitis was defined as inflammation or ulceration of the appendix or periappendix without obvious signs of necrosis or perforation [18]. Complicated appendicitis was defined as appendiceal inflammation with signs of gangrene or a perforation, large intraperitoneal infiltration, or abscess [18]. In case of discrepancy between intraoperative and histopathological findings, surgical findings were decisive, with the exception of malignancies as found at pathology examination.

The purpose of this study was not to diagnose appendicitis, but to estimate the severity of the appendicitis in patients diagnosed at the ED with acute appendicitis. Therefore, intraoperative finding of a normal appendix was categorized under the heading of uncomplicated appendicitis, whereas any urgent disease other than complicated appendicitis in need of surgery was categorized under complicated appendicitis, including appendiceal malignancy.

### Outcomes

The primary outcome of this study was the accuracy of the doctor’s final judgement — based on subjective interpretation of clinical, laboratory, and imaging results — for the diagnosis of complicated appendicitis in terms of sensitivity, specificity, positive predictive value (PPV), and NPV. The secondary outcome was the accuracy of the radiologist for the diagnosis of complicated appendicitis based on imaging. Additionally, all assessment outcomes were stratified for years of experience and level of certainty of the judgement. The outcomes were stratified for doctor’s field of expertise and whether or not they consulted a surgeon; radiology outcomes for imaging modality.

### Statistical analyses

Normally distributed data were shown as mean with standard deviation (SD) and non-normally distributed data as median with the interquartile range (IQR). Statistical significance was considered with a *p*-value < 0.05. Inter-observer agreement of diagnosis between doctors and radiologists was expressed as Cohen’s *κ* coefficient. This coefficient was interpreted as follows: ≤ 0.20 as none to slight agreement, 0.21–0.40 as fair, 0.41–0.60 as moderate, 0.61–0.80 as substantial, and 0.81–1.00 as (almost) perfect. Statistical analyses were performed using SPSS® version 26.0.

## Results

**Fig. 1:**
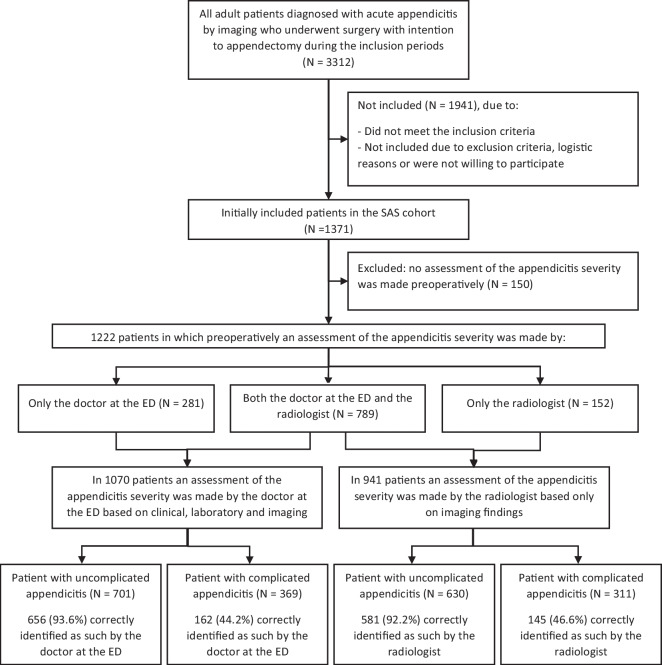
Flowchart of included patients

A total of 1371 adult patients with an imaging-confirmed diagnosis of acute appendicitis who underwent surgery with the intention to appendectomy were included in the original prospective SAS cohort (Fig. 1, flowchart). In 1222 patients, diagnostic assessment of the appendicitis severity was made. In 1070 patients, this assessment was made by the doctor at the ED based on clinical, laboratory, and imaging findings, and in 941 patients by the radiologist based on only imaging findings. For 789 patients, both the emergency doctor and the radiologist assessed the appendicitis severity preoperatively.

### Patients’ characteristics and reference standard diagnosis

Patients’ characteristics are summarized in Table 1. More than half of the patients were diagnosed with ultrasound (US) as only imaging modality (62.1%). According to the reference standard, 805 (65.9%) patients had a non-urgent disease, of which 790 (64.6%) had uncomplicated appendicitis and 15 (1.2%) a normal appendix. In comparison, overall, 417 (34.1%) patients had an urgent disease, of which 150 (12.3%) had gangrenous and 228 (18.7%) perforated appendicitis. In 21 (1.7%) patients, a malignancy was found. Baseline characteristics of patients with uncomplicated versus complicated appendicitis are described in Table S2.

**Table 1:** Baseline characteristics of 1222 patients with an assessment of the appendicitis severity by the emergency department doctor and/or radiologist

Characteristic	Total cohort (*n* = 1222)
Age, median (IQR), years	41 (28–56)
Female sex, no./total no. (%)	592 (48.4)
Temperature, mean (SD), in °C	37.2 (0.8)
Duration of symptoms (%)	
1 day (0**–**24 h)	573 (46.9)
2 days (24**–**48 h)	363 (29.7)
3 days or more (> 48 h)	286 (23.4)
WBC count, mean (SD), 10^9^/L	13.4 (4.5)
CRP, median (IQR), mg/L	50 (21–97)
Diagnostic imaging, no./total no. (%)	
US	759 (62.1)
CT	454 (37.2)
MRI	9 (0.7)
Final diagnosis, *n* (%)	
Uncomplicated appendicitis/disease	805 (65.9)
Normal appendix	15 (1.2)
Uncomplicated appendicitis	790 (64.6)
Complicated appendicitis/disease	417 (34.1)
Gangrenous appendicitis	150 (12.3)
Perforated appendicitis	228 (18.7)
Appendicitis with abscess/infiltrate/other*	39 (3.1)

*C*, Celsius; *CRP*, C-reactive protein; *CT*, computed tomography; *IQR*, interquartile range; *MRI*, magnetic resonance imaging; *SD*, standard deviation; *US*, ultrasound; *WBC*, white blood cell count

^*^(Suspected for) malignancy based on intraoperative findings or any other urgent disease

### Assessment at the emergency department

The assessment of “uncomplicated” or “complicated” appendicitis at the ED was by and large made by doctors from the surgical department (70.0%) or emergency doctors (25.9%); 4.1% missing data. The majority were novice doctors with 0–1 (45.2%) or 1–3 (31.1%) years of experience, whereas 8.3% had 3–5 years of experience and 9.9% even more than 5 years; 5.4% missing data. About half of the assessments of appendicitis severity were made in consultation with the surgeon on call (48.3%). The majority (81.1%) of the assessments were a “certain” decision, meaning 7 points or higher in certainty on an 11-point Likert scale (0 to 10), with a median certainty of 8 (IQR 7–9). In 14.2% of patients, the assessment was “uncertain,” and in 4.7%, this variable was missing.

At the ED, the severity of acute appendicitis was assessed by the attending doctor as “uncomplicated” in 862 patients (80.6%) versus “complicated” appendicitis in 208 patients (19.4%). Overall, these judgements were correct in 76.4%. Almost all patients with true uncomplicated appendicitis were accurately identified (656 of 701 (93.6%)), in contrast to only 163 of 369 (44.2%) patients with true complicated appendicitis (Fig. 1). This resulted in a sensitivity of 44.2%, a specificity of 93.6%, a PPV of 78.4%, and an NPV of 76.1% for the final doctor’s judgement of complicated appendicitis (Table 2).

**Table 2:** Accuracy of appendicitis severity as assessed by the doctor at the emergency department

Assessment at ED	*n* = 1070	Sensitivity	Specificity	PPV	NPV
All patients		44.2	93.6	78.4	76.1
Assessment after consultation of the surgeon on call	*n* = 985/1070				
With consultation (%)	517 (52.5)	47.2	95.0	85.2	74.8
Without consultation (%)	468 (47.5)	39.2	93.2	71.8	77.7
Certainty, based on a 11-point Likert scale (0**–**10)	*n* = 1020/1070				
Certain, meaning 7/10 or higher (%)	868 (85.1)	43.1	96.1	85.6	75.8
Uncertain**.** meaning 6/10 or lower (%)	152 (14.9)	43.2	82.4	50.0	78.1
Years of experience of the doctor at the ED	*n* = 1022/1070				
≤ 1 year (%)	484 (47.8)	44.8	94.4	80.2	77.1
1**–**3 years (%)	333 (32.9)	38.9	91.4	69.8	74.4
3**–**5 years (%)	89 (8.8)	46.7	91.5	73.7	77.1
> 5 years (%)	106 (10.5)	42.4	97.3	87.5	78.9
Type of doctor at the ED	*n* = 1026/1070				
Surgical trainee	741 (72.2)	44.9	93.4	76.8	77.6
Consultant surgeon	8 (0.8)	66.7	100.0	100.0	83.3
Emergency medicine trainee	205 20.0)	35.1	94.5	79.4	70.8
Emergency medicine consultant	60 (5.8)	43.5	100.0	100.0	74.0

*ED*, emergency department; *NPV*, negative predictive value; *PPV*, positive predictive value

Severity assessments that were indicated as “certain” scored higher in specificity and PPV than those of lower certainty (Table 2). Furthermore, doctors with more than 5 years of experience achieved the highest specificity and PPV for complicated appendicitis; comparable for severity accuracy among patients who had been consulted by the surgeon on call. Counterintuitively, doctors with ≤ 1 year experience had higher specificity than those with 1–5 years of experience. Finally, no differences were observed between surgery and emergency medicine doctors, although consultant doctors scored better than trainees, in particular for PPV of complicated appendicitis (Table 2). Sensitivity exceeded 50% only in the eight patients who had been assessed directly by the consultant surgeon.

### Assessment by the radiologist

For the 941 patients with a registered appendicitis severity assessment by the radiologist, US was the imaging modality in 615 (65.4%), CT in 320 (34.0%), and MRI in 6 patients (0.6%). Differentiation between “uncomplicated” and “complicated” appendicitis was made by radiology trainees in 218 patients (23.2%), by consultant general radiologists in 578 patients (61.4%), and by consultant abdominal radiologists in 101 patients (10.7%). The vast majority of assessments, for which a level of certainty was indicated, were rated as “certain” (716 of 740; 96.8%).

Based on the radiologist’s assessment, 747 of 941 patients (79.4%) were classified as having uncomplicated appendicitis and 194 (20.6%) patients as complicated appendicitis. These severity assessments were correct in 77.2% when compared to the reference standard. Almost all patients with true uncomplicated appendicitis (581 of 630 (92.2%)) but only 145 of 311 (46.6%) patients with true complicated appendicitis were accurately classified. This results in a sensitivity of 46.6%, a specificity of 92.2%, a PPV of 74.7%, and a NPV of 77.8% for radiologist’s final imaging judgement of complicated appendicitis (Table S1).

The proportion of patients with complicated appendicitis in the US subgroup was 24.6% compared to 49.4% in the CT subgroup. In patients with US imaging, sensitivity, specificity, PPV, and NPV were 39.1%, 91.6%, 60.2%, and 82.2%, respectively, versus 53.2%, 93.8%, 89.4%, and 67.3% for patients with CT imaging.

### Inter-observer agreement

In 789 patients, both the attending doctor at the ED and the radiologist made an assessment for appendicitis severity preoperatively. These estimates were in agreement between both assessors in 91.3% of the cases, with a substantial inter-observer agreement (Cohen’s *κ* = 0.73). Also, a substantial agreement was found within the subgroups of patients diagnosed with US or CT (Cohen’s *κ* = 0.70 and 0.74, respectively).

### Patient characteristics of correct vs incorrect severity assessments

Among 369 patients with complicated appendicitis according to the reference standard, 163 (44.2%) were correctly identified as such at the ED. These correctly classified patients were significantly older than patients wrongfully classified as having uncomplicated appendicitis; the median age was 54 (IQR 38–65) compared to 49 years (IQR 37–60), respectively (Table 3). Moreover, patients correctly identified as complicated appendicitis had higher CRP levels (median 139 (IQR 70–217) versus 69 mg/L (IQR 35–134), respectively) and longer duration of symptoms before presentation (proportion of patients with complaints ≥ 3 days: 50.9% vs 23.3%, respectively) than those wrongfully classified.

**Table 3:** Characteristics of all 369 patients with complicated appendicitis as reference standard diagnosis assessed by the doctor at the ED: correct vs incorrect severity assessment

	Correctly identified as complicated appendicitis (*n* = 163)	Wrongfully labelled as uncomplicated appendicitis (*n* = 206)	*p*-value
Age, median (IQR), years	54 (38–65)	49 (37–60)	0.010
Age in years (%)			0.024
18**–**30	27 (16.6)	37 (18.0)	
31**–**60	76 (46.6)	123 (59.7)	
> 60	60 (36.8)	46 (22.3)	
Female sex, no./total no. (%)	59 (36.2)	79 (38.3)	0.671
ASA, no./total no. (%)			0.059
1	55/144 (38.2)	83/170 (48.8)	
2**–**4	89/144 (61.8)	87/170 (51.2)	
Temperature, mean (SD), in °C	37.6 (0.9)	37.4 (0.8)	0.097
Duration of symptoms (%)			> 0.001
1 day (0**–**24 h)	34 (20.9)	93 (45.1)	
2 days (24**–**48 h)	46 (28.2)	65 (31.6)	
3 days or more (> 48 h)	83 (50.9)	48 (23.3)	
CRP, median (IQR), mg/L	139 (70–217)	69 (35–134)	> 0.001
WBC count, mean (SD), 10^9^/L	14.5 (4.8)	14.6 (4.3)	0.914

*ASA*, American Society of Anesthesiologists; *C*, Celsius; *CRP*, C-reactive protein; *ED*, emergency department; *IQR*, interquartile range; *SD*, standard deviation; *WBC*, white blood cell count

## Discussion

This large, prospective, multicenter study demonstrated that final diagnostic judgements of doctors at the ED, integrating all available clinical, laboratory, and imaging results, largely underestimate the number of patients with complicated appendicitis. More than half of all patients with true complicated appendicitis was incorrectly classified as uncomplicated appendicitis according to these judgements, even if these decisions were marked as “certain” by the assessors. Similar results were seen among radiologists who based their diagnostic judgement on imaging alone. Ruling out complicated appendicitis on final doctor’s judgement or final imaging interpretation remains unreliable for the selection of patients with uncomplicated appendicitis who may be treated without surgery.

Differentiation between complicated and uncomplicated acute appendicitis is important for the choice of treatment. Several large trials have shown that antibiotic treatment of uncomplicated appendicitis in adults is effective and safe [2–5]. It is important to exclude complicated appendicitis when selecting patients eligible for antibiotic treatment. A NPV of 75.8% in patients with a “certain” judgement of uncomplicated appendicitis is equivalent to 24.2% of patients who were thought to have “uncomplicated appendicitis” based on a confident doctor’s judgement but turned out to have “complicated appendicitis.” The studies that randomized between antibiotics and surgery used objective inclusion criteria. Within the patients randomized for surgery, they described a proportion of patients with complicated appendicitis of 1.5–18% [2, 3]. This means that objectively measurable variables work better than subjective judgement for making an accurate assessment of appendicitis severity.

Several objective variables are known to be predictive for differentiation between complicated and uncomplicated appendicitis, e.g., age [19] and CRP levels [20]. These characteristics indeed differ significantly between patients with uncomplicated and complicated appendicitis in our cohort (Table S2). When comparing patients with complicated appendicitis who were correctly identified at ED by the doctor to patients wrongfully classified as having uncomplicated appendicitis, these previously published distinctive variables (age and CRP level) differ significantly. This means that indeed these variables contributed, consciously or unconsciously, to the doctor’s final diagnostic judgement. Moreover, the doctor’s interpretation of objective findings decreases the sensitivity of these distinctive variables for ruling out complicated appendicitis. This means that adding the subjective judgement does not guarantee more accurate identification of complicated appendicitis. Prior to this study, we hoped that subjective aggregated assessment would add value in patients in whom objective variables were deficient in differentiation of appendicitis severity. Unfortunately, this does not appear to be true.

Only a small proportion of patients were assessed by a consultant surgeon (0.8%) or an emergency medicine consultant (5.8%). The accuracy of these consultant assessments surpassed that of other doctors, with sensitivities of 43.5–66.7% vs 35.1–44.9% and specificities of 100.0 vs 93.4–94.5%, respectively. This suggests that overall accuracy could increase if the risk assessments were conducted by consultants for all patients. However, a prior study, in which both surgical trainees and consultant surgeons made an estimate of the diagnosis in all patients with acute abdominal pain, revealed that the diagnostic accuracy of clinical assessments does not improve when a surgeon, rather than a surgical trainee, conducts the assessments [21].

Although sensitivity and NPV did not achieve high values, a high specificity was found. This means that the false positive rate of the subjective diagnostic judgement of the doctor at the ED is low. Unfortunately, this has limited clinical utility. When treatment options must be considered, it is precisely the exclusion of complicated appendicitis that is crucial, meaning that sensitivity and NPV are important rather than specificity and PPV.

In most patients, both the radiologist and the doctor at the ED made an assessment for “uncomplicated” or “complicated” acute appendicitis. There was a substantial inter-observer agreement among cases. A possible explanation for this is that the doctor at the ED, integrating available findings including imaging results, likely was influenced by the radiologist’s final assessment based on imaging that was stored in the electronic patient record.

The reference standard used in this study slightly deviated from the previously published study protocol [15]. Initially, any sign of gangrene on histopathology led to a diagnosis of complicated appendicitis. However, advancing insights led to a more prominent role for the intraoperative findings when these differed from histopathology results in case of microscopic signs of necrosis without any macroscopic necrosis as clinical relevance is unclear.

## Limitations

The study protocol has been published before the final analysis was executed and all predesigned analyses were performed [15]. This study was performed prospective and multicenter. All patients underwent diagnostic imaging and thereby had imaging-confirmed acute appendicitis. For each included patient, a reference standard from an adjudication committee was available. For most included patients, both the estimate of the doctors at the ED and radiologist’s final diagnostic judgement were available. Apart from these strengths, the study has several limitations. First, the present study describes an analysis of patients included in the SAS study [15], with the primary aim to validate the Atema score [9], which is an objective scoring model for appendicitis severity. Although the individual patient’s score result had not been made available during the runtime of the study, the doctors who estimated the appendicitis severity were aware of the included objective variables, which may have led to their judgement being influenced by conscious or subconscious emphasis on the results of those variables. Second, as shown in the flowchart, more than half of the eligible patients were not included, which may have contributed to a relatively high rate of complicated appendicitis and may have affected the results in other ways. Although clearly stated in the questionnaire, the question about level of certainty of diagnosis may have been wrongly interpreted as certainty of the very diagnosis of acute appendicitis and not so much the specification “uncomplicated” or “complicated.” Moreover, this level of certainty of diagnosis, like other variables, was noted in the electronic patient record to which patients themselves have access when they register to “Mychart.” This may have led doctors to prefer not to indicate doubt and fill in higher level of certainty than they felt.

## Conclusions

More than half of all patients with true complicated appendicitis is incorrectly classified as uncomplicated appendicitis according to the final diagnostic judgements of doctors at the ED, integrating all available clinical, laboratory, and imaging results. Comparable accuracy is found for radiologists assessing diagnostic imaging. Thereby, these diagnostic judgements are not sufficiently reliable in ruling out complicated appendicitis. With respect to selection of patients with true uncomplicated appendicitis for antibiotic treatment without appendectomy, subjective final judgements of doctors at the emergency department or radiologists’ interpretation of imaging results are still far from perfect. Scoring systems for appendicitis severity that compile results from objective variables into a final probability of complicated appendicitis among patients with acute appendicitis, as a percentage with a confidence interval, may improve accuracy of severity assessment.

### Supplementary Information

Below is the link to the electronic supplementary material.

Supplementary file1 (DOCX 18 KB)

Supplementary file2 (DOCX 14 KB)

Supplementary file3 (DOCX 14 KB)
