# Supplementary material for: Diagnostic accuracy of doctors at the emergency department and radiologists in differentiating between complicated and uncomplicated acute appendicitis
Source: Eur J Trauma Emerg Surg. 2024 Jan 17;50(3):837–45. doi: 10.1007/s00068-023-02442-2 (PMC11249706; doi:10.1007/s00068-023-02442-2)
Supplement: Supplementary file 1 — Supplementary file1 (DOCX 18 KB) [file 68_2023_2442_MOESM1_ESM.docx]

**Supplementary materials**

**Authors**: JCG Scheijmans, WJ Bom, RS Deniz, AAW van Geloven, MA Boermeester; SAS Collaborative Group
**The SAS Collaborator group**: F Alberts (1); S Bachiri (2); MA den Bakker (3); B Bisschops (4); E Boersma (5); MDM Bolmers (6); WM Bosman(7); H Bril (8); C Buurman (9); EFW Courrech Staal (10); P Davids (11); R Detering (13); MGW Dijkgraaf (14); BL Dijkstra (15); P Drillenburg (16); A Dinaux (7); P van Duijvendijk (17); WJ van Eden (13); R Franken (18); S Gans (17); M Gaspersz (19); AM van Geel (20); MF Gerhards (13); H Ghori (12); JWC Gratama (21); I Groenendijk (19); P Hellebrekers (10); A van Hemert (18); M Henebiens(1); H Heydari (12); K in ’t Hof (22); TJ Hoogteijling (18); PM Huisman (23); G van Ingen (24); S Jensch (25); AM Jonker (26); F Joosten (27); L Koens (28); N Kraaijvanger (29); KC Kuijpers (30); TYS Le Large (6); D Linzel (31); MHJ Loos (17); AMF Lopes Cardozo (2); LB Meijer-Jorna (32); M Mulder (33); N Mullaart (15); SJ Oosterling (18); J Oudejans (34); T Pappot (35); S Peeters (31); C Pleiter (36); MA de Roos (5); C Rosman (37); CC van Rossem (19); MM Scheurkogel (38); L Scholten (22); [T Schut](mailto:t.schut@gelre.nl) (9); J Stoker (39, 40); OWT Tiddens (41); S Ubels (37); FEE de Vries (7); LFJ Walraven (42); EMA Wiegerinck (25); JK Wiggers (13); M Witt (43); N Wolfhagen (12); L van de Wouw (44).

Affilitations:

1. Spaarne Gasthuis, Department of Radiology, Haarlem and Hoofddorp, The Netherlands
2. Noordwest Hospitalgroup, Department of Surgery, Alkmaar, The Netherlands
3. Maasstad Hospital, Department of Pathology, Rotterdam, The Netherlands
4. Albert Schweitzer Hospital, Department of Radiology, Dordrecht, The Netherlands
5. Rijnstate Hospital, Department of Surgery, Arnhem, The Netherlands
6. Dijklander Hospital, Department of Surgery, Hoorn, The Netherlands
7. Albert Schweitzer Hospital, Department of Surgery, Dordrecht, The Netherlands
8. Spaarne Gasthuis, Department of Pathology, Haarlem and Hoofddorp, The Netherlands
9. Gelre Hospitals, Department of Emergency Medicine, Zuthpen, The Netherlands
10. Maasstad Hospital, Department of Radiology, Rotterdam, The Netherlands
11. Diakonessenhuis, Department of Surgery, Utrecht, The Netherlands
12. Amsterdam University Medical Center, Department of Surgery, University of Amsterdam, Amsterdam, The Netherlands
13. OLVG, Department of Surgery, Amsterdam, The Netherlands
14. Amsterdam University Medical Center, Department of Epidemiology and Data Science, University of Amsterdam, Amsterdam, The Netherlands
15. Dijklander Hospital, Department of Emergency Medicine, Hoorn, The Netherlands
16. OLVG, Department of Pathology, Amsterdam, The Netherlands
17. Gelre Hospitals, Department of Surgery, Apeldoorn, The Netherlands
18. Spaarne Gasthuis, Department of Surgery, Haarlem and Hoofddorp, The Netherlands
19. Maasstad Hospital, Department of Surgery, Rotterdam, The Netherlands
20. Noordwest Hospitalgroup, Department of Radiology, Alkmaar, The Netherlands
21. Gelre Hospitals, Department of Radiology, Apeldoorn, The Netherlands
22. Flevohospital, Department of Surgery, Almere, The Netherlands
23. Tergooi Medical Center, Department of Radiology, Hilversum, The Netherlands
24. Regional Pathology Laboratory PAL, Dordrecht, The Netherlands
25. OLVG, Department of Radiology, Amsterdam, The Netherlands
26. Gelre Hospitals, Department of Pathology, Apeldoorn, The Netherlands
27. Rijnstate Hospital, Department of Radiology, Arnhem, The Netherlands
28. Amsterdam University Medical Center, Department of Pathology, University of Amsterdam, Amsterdam, The Netherlands
29. Rijnstate Hospital, Department of Emergency Medicine, Arnhem, The Netherlands
30. Diakonessenhuis, Department of Pathology, Utrecht, The Netherlands
31. Flevohospital, Department of Emergency Medicine, Almere, The Netherlands
32. Noordwest Hospitalgroup, Symbiant, Department of Pathology, Alkmaar, The Netherlands
33. Dijklander Hospital, Department of Pathology, Hoorn, The Netherlands
34. Tergooi Medical Center, Department of Pathology, Hilversum, The Netherlands
35. Diakonessenhuis, Department of Radiology, Utrecht, The Netherlands
36. Flevohospital, Department of Radiology, Almere, The Netherlands
37. Radboud Medical Center, Department of Surgery, University Medical Center Nijmegen, Nijmegen, The Netherlands
38. Dijklander Hospital, Department of Radiology, Hoorn, The Netherlands
39. Amsterdam UMC location University of Amsterdam, Department of Radiology and Nuclear Medicine, Meibergdreef 9, 1105 AZ Amsterdam, The Netherlands
40. Amsterdam Gastroenterology Endocrinology and Metabolism, Amsterdam, The Netherlands
41. OLVG, Department of Emergency Medicine, Amsterdam, The Netherlands
42. Diakonessenhuis, Department of Emergency Medicine, Utrecht, The Netherlands
43. Spaarne Gasthuis, Department of Emergency Medicine, Haarlem and Hoofddorp, The Netherlands
44. Tergooi Medical Center, Department of Surgery, Hilversum, The Netherlands

**Author contributions**

**Concept and design**: Scheijmans, Bom, van Geloven, Boermeester.

**Acquisition of funding**: Bom, van Geloven, Boermeester.

**Acquisition of data**: Scheijmans, Bom, Deniz.

**Adjudication committee (data interpretation)**: Scheijmans, Bom, van Geloven, Boermeester.

**Analysis of data**: Scheijmans, Bom, van Geloven, Boermeester.

**Drafting the article**: Scheijmans, Bom, Deniz, van Geloven, Boermeester.

**Revising the article**: Scheijmans, Bom, Deniz, van Geloven, Boermeester.

**Final approval of the version published**: Scheijmans, Bom, Deniz, van Geloven, Boermeester.
