# Supplementary material for: Diagnostic accuracy of doctors at the emergency department and radiologists in differentiating between complicated and uncomplicated acute appendicitis
Source: Eur J Trauma Emerg Surg. 2024 Jan 17;50(3):837–45. doi: 10.1007/s00068-023-02442-2 (PMC11249706; doi:10.1007/s00068-023-02442-2)
Supplement: Supplementary file 2 — Supplementary file2 (DOCX 14 KB) [file 68_2023_2442_MOESM2_ESM.docx]

| Table S1. Accuracy of appendicitis severity as assessed at radiology | | | | | |
| --- | --- | --- | --- | --- | --- |
| Assessment at Radiology | Number of patients | Sensitivity | Specificity | PPV | NPV |
| All | 941* | 46,6 | 92,2 | 74,7 | 77,8 |
| US | 615**^ⴕ^** | 39,1 | 91,6 | 60,2 | 82,2 |
| Radiology trainee | 158 | 40,5 | 94,0 | 70,8 | 81,3 |
| Consultant Radiologist | 355 | 39,5 | 90,0 | 55,7 | 82,3 |
| Consultant Radiologist, specialized in the abdomen | 66 | 37,5 | 94,0 | 66,7 | 82,5 |
| CT | 320**^◊^** | 53,2 | 93,8 | 89,4 | 67,3 |
| Radiology trainee | 57 | 61,3 | 88,5 | 86,4 | 65,7 |
| Consultant Radiologist | 222 | 50,5 | 94,1 | 88,1 | 68,7 |
| Consultant Radiologist, specialized in the abdomen | 35 | 55,0 | 100,0 | 100,0 | 62,5 |
| CT, computed tomography; NPV, negative predictive value; PPV, positive predictive value; US, ultrasound. * In six patients (0.6%) in MRI was performed. ^ⴕ^ 14 patients were assessed by a radiology technician, in 22 patients info about the assessor was missing. ^◊^ In six patients info about the assessor was missing. | | | | | |
