# Supplementary material for: Diagnostic accuracy of doctors at the emergency department and radiologists in differentiating between complicated and uncomplicated acute appendicitis
Source: Eur J Trauma Emerg Surg. 2024 Jan 17;50(3):837–45. doi: 10.1007/s00068-023-02442-2 (PMC11249706; doi:10.1007/s00068-023-02442-2)
Supplement: Supplementary file 3 — Supplementary file3 (DOCX 14 KB) [file 68_2023_2442_MOESM3_ESM.docx]

| Table S2. Characteristics of all patients presented according to their final diagnosis: uncomplicated appendicitis vs complicated appendicitis | | |
| --- | --- | --- |
| Characteristic | Uncomplicated appendicitis (n = 805) | Complicated appendicitis (n = 417) |
| Age, median (IQR), years | 37 (26-51) | 51 (36-62) |
| Female sex (%) | 429 (53.3) | 163 (39.1) |
| Temperature, mean (SD), in °C | 37.0 (0.7) | 37.4 (0.9) |
| Duration of symptoms (%) |  |  |
| 1 day (0-24 hours) | 432 (53.7) | 141 (33.8) |
| 2 days (24-48 hours) | 238 (29.6) | 125 (30.0) |
| 3 days or more (> 48 hours) | 135 (16.8) | 151 (36.2) |
| WBC count, mean (SD), 10^9/L | 12.9 (4.3) | 14.4 (4.6) |
| CRP, median (IQR), mg/L | 37 (16-67) | 96 (47-177) |
| Diagnostic imaging (%) |  |  |
| US | 565 (70.2) | 194 (46.5) |
| CT | 233 (28.9) | 221 (53.0) |
| MRI | 7 (0.9) | 2 (0.5) |
| Abscess at pre-treatment imaging (%) | 4 (0.5)* | 35 (8.4) |
| C, Celsius; CRP, C-reactive protein; CT, computed tomography; IQR, interquartile range; MRI, magnetic resonance imaging; SD, standard deviation; US, ultrasound; WBC, white blood cell count.  * No abscess was found in these four patients during surgery | | |
